# A detailed comparison of analysis processes for MCC-IMS data in disease classification—Automated methods can replace manual peak annotations

**DOI:** 10.1371/journal.pone.0184321

**Published:** 2017-09-14

**Authors:** Salome Horsch, Dominik Kopczynski, Elias Kuthe, Jörg Ingo Baumbach, Sven Rahmann, Jörg Rahnenführer

**Affiliations:** 1 Department of Statistics, TU Dortmund University, Dortmund, Germany; 2 Bioinformatics, Computer Science XI, TU Dortmund University, Dortmund, Germany; 3 Genome Informatics, Institute of Human Genetics, University of Duisburg-Essen, University Hospital Essen, Essen, Germany; 4 Faculty of Applied Chemistry, Reutlingen University, Reutlingen, Germany; University of Connecticut, UNITED STATES

## Abstract

**Motivation:**

Disease classification from molecular measurements typically requires an analysis pipeline from raw noisy measurements to final classification results. Multi capillary column—ion mobility spectrometry (MCC-IMS) is a promising technology for the detection of volatile organic compounds in the air of exhaled breath. From raw measurements, the peak regions representing the compounds have to be identified, quantified, and clustered across different experiments. Currently, several steps of this analysis process require manual intervention of human experts. Our goal is to identify a fully automatic pipeline that yields competitive disease classification results compared to an established but subjective and tedious semi-manual process.

**Method:**

We combine a large number of modern methods for peak detection, peak clustering, and multivariate classification into analysis pipelines for raw MCC-IMS data. We evaluate all combinations on three different real datasets in an unbiased cross-validation setting. We determine which specific algorithmic combinations lead to high AUC values in disease classifications across the different medical application scenarios.

**Results:**

The best fully automated analysis process achieves even better classification results than the established manual process. The best algorithms for the three analysis steps are (i) SGLTR (Savitzky-Golay Laplace-operator filter thresholding regions) and LM (Local Maxima) for automated peak identification, (ii) EM clustering (Expectation Maximization) and DBSCAN (Density-Based Spatial Clustering of Applications with Noise) for the clustering step and (iii) RF (Random Forest) for multivariate classification. Thus, automated methods can replace the manual steps in the analysis process to enable an unbiased high throughput use of the technology.

## Introduction

In many medical applications diagnosing diseases requires complex examinations, which can be expensive, time consuming and painful for the patient. Therefore, alternatives like analyzing breath gas should be considered. Breath gas of a person is permanently available and can be collected non-invasively which makes data collection a gentle examination. Furthermore, it has already been shown that human breath is very informative regarding processes inside the body [1, 2], since gas exchange in the lungs enables inference on the blood compound. The analysis of blood may require growing cultures, which can consume several hours or days. Using a technique like MCC-IMS (multi capillary column—ion mobility spectrometry), a full analysis of human breath can be accomplished in approximately 10 minutes. It does not require many consumable supplies, making it rather cheap. MCC works at ambient pressure. In particular, it does not require a vacuum pump system, allowing minimization of the entire device. Therefore, it is also suitable for mobile applications where immediate evaluation of a sample is important. Pre-concentration steps are unnecessary.

For statistical classification, as well as for biological interpretability, the definition of variables, so called features, is inevitable. The task is to identify the peaks which can be visually spotted in the image. This task is called *peak picking*. The current gold standard requires manual supervision of this step, which is time consuming and expensive. This is an obstacle for the technology regarding broad application and immediate evaluation in the field. In this work we compare six alternatives in order to find an algorithm that completes the task fully automatically, quicker and with results as good as or even better than the current manual gold standard.

Once the peaks in a single measurement (from one experiment) are found, methods are required that align peak locations between several measurements (e.g. from different people). We call this step *peak clustering* since it can be seen as a statistical clustering task, grouping locations from peaks across experiments. Peak positions of the peaks belonging to a certain cluster are merged to one peak representing one metabolite. In the context of the subsequent classification, the clusters then represent the features. The result of peak clustering is a dataset with an a priori unknown number of metabolites (clusters). In this study, we compare five different peak clustering algorithms to the semi-manual gold standard that combines peak picking and peak clustering in one step.

We combine all combinations of algorithms for peak picking and peak clustering that are technically feasible and evaluate all methods on three different datasets, extending a preliminary study [3]. For each dataset, the statistical classification problem is the discrimination between two groups of patients. One has a certain disease, and the other is a healthy control. We select six popular algorithms, that have proven to be effective in many other tasks, out of the large number of approaches for binary classification.

We evaluate all combinations of peak picking, peak clustering and classification by their classification performance. This means that peak picking or peak clustering methods are considered favorable, when the ability to distinguish healthy from diseased people based on these peaks exceeds the ability of other methods. In order to avoid overfitting and thus biased results, we apply a cross-validation scheme to assess the quality of classification results, and nested cross-validation when parameter optimization is performed in a classification algorithm. We repeat this process 50 times to observe the variability in the results. We use classification AUC values for the evaluation. Note that peak picking and clustering were not performed inside the cross-validation due to the extremely extended effort for a manual peak detection in each replication of each cross-validation-run.

## Data

### Datasets

The first dataset arises from investigations at the lung clinic Hemer (official clinical trial, Clinical Trials Identifier: NCT00632307), where 10 mL exhaled air of 92 patients suffering COPD at different stages and 35 healthy controls was investigated using a BreathDiscovery MCC/IMS of B&S Analytik GmbH, Dortmund, Germany. Preliminary results were published by [4]. The clinical trial is in progress until 2020 and was approved by the ethics committee of the university of Münster (DIMDI). Our dataset contains measurements from 2006/2007.

The second dataset contains measurements on exhaled breath from 30 patients whose airways are either infected or colonized by *Pseudomonas aeruginosa*, and from 37 healthy non-smoker controls. Patients were recruited from the Department of Pulmonology, Ruhrlandklinik, University Hospital of Essen, Germany in 2010. Healthy controls were employees of the hospital. The study was approved by the ethics committee of the university of Essen, with informed consent of all subjects. On a similar dataset, [5] identified single peaks with differential intensities between the two groups.

The third dataset was obtained from the Knappschaftkrankenhaus Dortmund under the local ethics committee agreement in 2012. Here about 10 mL of exhaled air from 39 patients suffering from asbestosis were taken during regularly standard investigations related to pension requirements and of exhaled air from 30 healthy controls.

In all studies, written consent according to the instructions of the ethics committees was obtained. No medical studies were performed explicitly for this paper.

### Technical devices

**Fig 1:**
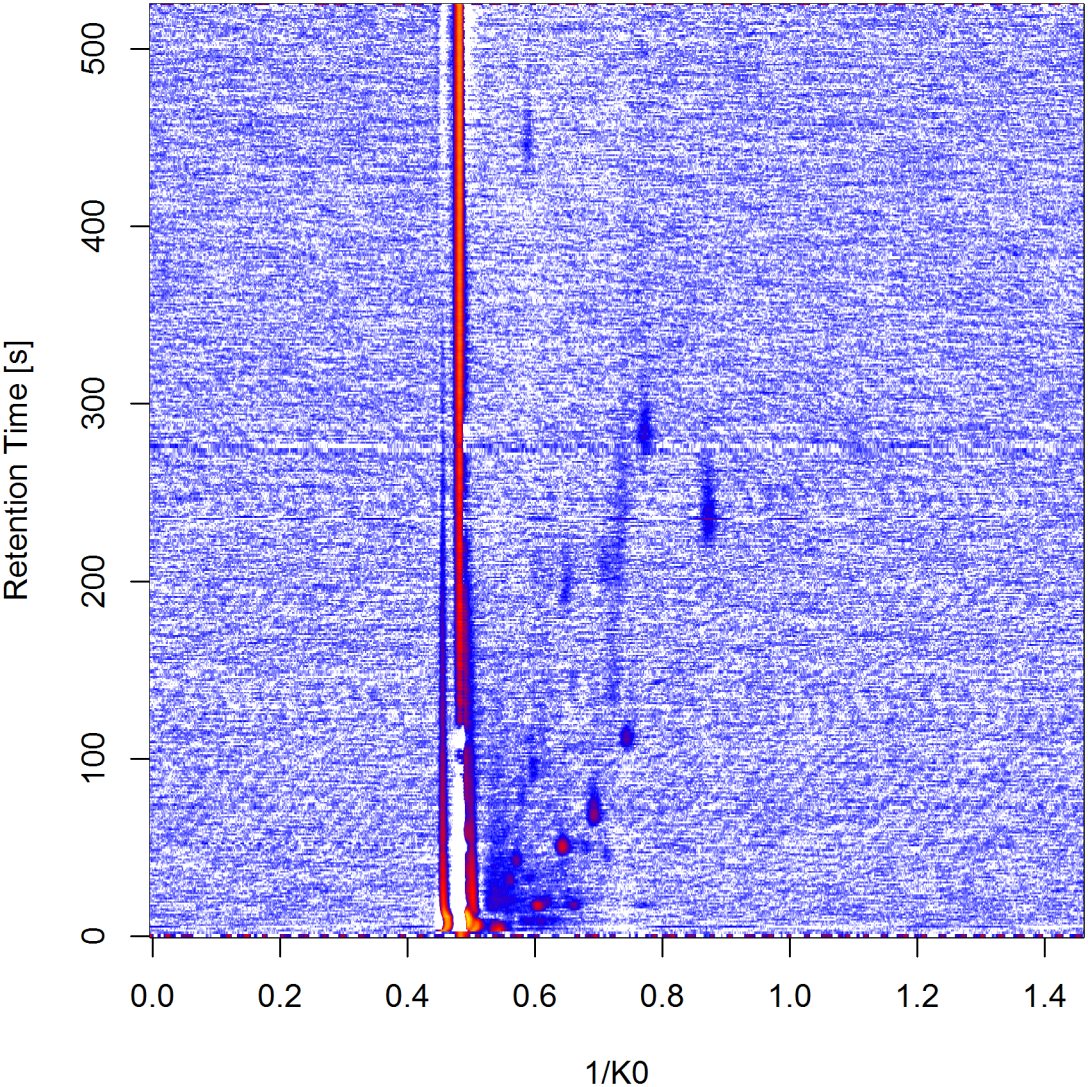
Example for a raw measurement from the first dataset. The rows of the heatmap represent the retention times and the columns represent 1/K0, a transformation of the drift time. The colors display the signal intensities with increasing values from white over blue and red to yellow.

Breath gas analysis is accomplished by Multi capillary column—ion mobility spectrometry (MCC-IMS). For measuring one probe, the exhaled air first enters the MCC-part of the device for a pre-separation. It is operated at 40°C isotherm and at ambient pressure. Depending on physical and chemical characteristics, different molecules need specific times to pass through the column. These times of the molecules are measured and called retention times. Afterwards, these molecules are lead into the IMS part, where the molecules are ionized and lead through the drift tube, supported by an external electrical field. The time these ions take to pass the tube is measured and called drift time. The ions reach a Faraday plate and cause an electrical potential representing the amount of its corresponding metabolite in the breath sample. This so-called “‘intensity”’ is also recorded. Finally, three variables are measured to describe a molecule. The retention time and drift time characterize the metabolite. The intensity serves for quantification purposes. The three quantities (a transformation of the drift time (the inverse reduced mobility 1/K_0_), retention time and intensity) can be visualized together in a heatmap. An example of a raw measurement is displayed in Fig 1.

## Methods

The path from raw ion mobility measurements to classification requires three major steps each of which can be accomplished by various algorithms. In this section, all utilized algorithms are briefly introduced. Further information on the respective methods can be obtained from the given references. The three steps are:

1. Peak picking on single measurements including particular image filtering techniques
2. Peak clustering in order to determine, which peaks are the same across several measurements
3. Statistical classification using cross-validation.

We call the first two steps, peak picking and peak clustering, preprocessing. In total, 26 combinations of these two steps are considered. Each of the resulting datasets is passed to the six cross-validated classification analyses, resulting in 156 combinations of preprocessing and classification. The manual detection method combines peak picking and peak clustering within one step and is described in the section *Peak Clustering Methods*.

### Peak picking methods

In this section, we describe all evaluated automated peak picking methods. Each method yields a list of peak positions with corresponding signal intensities. The methods PME, OPME, SGTLR, and PDSA (see below) additionally provide parameters for the peak shapes, the first two use a statistical model, the others compute a bounding box.

Most peak detection methods employ two basic steps, see e.g. [6], namely smoothing and RIP (reactant ion peak) compensation. The RIP occurs from ionizing the carrier gas used (like synthetic air or pure nitrogen) and appears as an artifact on the raw image. It is compensated by estimating the shape of the RIP and subtracting it from the raw measurements. Smoothing and denoising are achieved with a fixed threshold, a low-pass filter or a smoothing kernel like the Savitzky-Golay filter [7].

We further distinguish between offline and online peak picking methods. Offline means that all data of a measurement are available for peak picking during the analysis process, whereas online methods scan through a measurement processing only single IM spectra or a small set of consecutive IM spectra that are afterwards directly discarded. In the latter case, not the whole raw data matrix has to be stored, which is favorable when working with resource-constrained embedded devices. *Automated detection in VisualNow*, *local maxima* (LM), and *peak model estimation* (PME) are offline methods, and *peak detection by slope analysis* (PDSA), *Savitzky-Golay Laplace-operator filter thresholding regions* (SGLTR), and *online peak model estimation* (OPME) are online methods.

#### Automated detection in VisualNow (VN^*a*^)

The commercial program VisualNow [8] contains an automated peak extraction method for MCC-IMS data, see [9] for details. *K*-means clustering is used for dividing cells of the data matrix (corresponding to image pixels) into peak and non-peak cells, based on intensity values. Afterwards, neighboring cells with label peak are merged to peak regions, and for each region the centroid is computed.

#### Local Maxima (LM)

LM is a method within the PEAX framework [10] that first identifies cells as candidate peaks, if their intensity exceeds a pre-specified threshold and the values of its eight neighbors. Close candidates are joined to peaks by a Weighted Cluster Editing algorithm (see *Cluster Editing* below). Position and intensity of a peak are obtained from the highest intensity cell.

#### Peak model estimation (PME)

PME [11] also starts with candidate peaks, but more sophisticated than LM, estimating smoothed derivatives in both directions of the data matrix. For grouping cells into peak regions an EM algorithm is used (see *EM Clustering* below). For determining position and intensity of a peak a seven-parameter model is fitted to the peak region by an EM-type algorithm.

#### Peak detection by slope analysis (PDSA)

PDSA [12] processes spectra (with fixed retention time, i.e. rows in the data matrix) one by one. First, segments with large intensities (higher than noise) are identified with a sliding window approach. Basically, a segment is kept when the sum of intensities within this area is a local maximum. Overlapping segments are discarded. Finally, segments are merged into peaks across neighboring retention time values, according to criteria described in [13].

#### Savitzky-golay laplace-operator filter thresholding regions (SGLTR)

SGLTR [12] searches for regions with high *curvature*. More precisely, we measure curvature by the sum of unmixed second derivatives in retention and drift time, known as the Laplace-operator. The Laplace-operator is approximated by a weighted Savitzky-Golay filter which is a local polynomial regression reducing the impact of noise. Peak locations are then identified as connected cells with filtered intensities above noise level.

#### Online peak model estimation (OPME)

OPME [14] is an online version of PME, based on a parametrization of the peak shape. For each spectrum (row of the data matrix), polynomials of degree two are fitted locally, describing one-dimensional candidate peak shapes. A global alignment approach is used to match shapes across retention time (spectra). Finally, also in retention direction (column of the data matrix), a polynomial of degree two is fitted to a matched group of peak candidates.

### Peak clustering methods

Peak clustering methods process the results of peak picking methods that were applied to a set of measurements, e.g. a cohort of patients and control group. The task is to determine cluster centers of peaks across measurements that may then represent the same analyte across samples. In the following, a *consensus peak* denotes a representative of such a cluster of peaks. The consensus peaks (position and intensity) are then the input for the classification methods described below. Therefore, a table with measurements and consensus peaks is provided.

In the following, we describe the manual annotation of peaks and four clustering methods, namely *Grid Squares*, *Density-Based Spatial Clustering of Applications with Noise*, *Cluster Editing*, and *EM Clustering*. The number of clusters must be determined by the algorithms, since the number of analytes is not known a priori.

#### Manual peak detection and clustering (VN^*m*^)

In the software VisualNow [8], peak detection and clustering are performed manually within one step. Interactively, rectangular regions are drawn on top of a heatmap visualization of a single data matrix from one measurement. The resulting region can be viewed across all measurements, and the regions can be manually adapted, added, or discarded. The final regions must not overlap, and the center of a peak (region) is determined by the highest intensity value in the region.

#### Automated clustering in VisualNow (VN^*a*^)

VisualNow uses a method based on *k*-means clustering to group peaks across measurements [15].

#### Grid squares (GS)

The overall region is partitioned into disjoint rectangular sub-regions. If at least a pre-specified number of peaks among the measurements falls into a rectangle, then the location of the consensus peak is defined as the average of all corresponding peak locations. The peak intensity of a consensus peak for a certain measurement is taken from the closest peak to the consensus peak location.

#### Density-based spatial clustering of applications with noise (DBSCAN)

DBSCAN [16] is a popular algorithm in data mining that iteratively searches for regions with many peaks across measurements. A random point is selected and all neighbors within a fixed distance are combined to a set. If the set size is at least minPts, the set is considered a peak cluster, and for new points in the cluster it is iteratively checked if they have at least minPts in their neighborhood. In this case these points are added to the cluster. When no more points can be added the cluster is closed. The next clusters are built in the same way, always starting with an unvisited random point. As consensus peaks we define the centroids of the final clusters.

#### Cluster editing (CE)

Weighted cluster editing [17, 18] constructs a graph that separates the peaks into disjoint cliques by insertion and deletion of edges. The distances between the peaks serve as weights (costs) for the optimization problem such that the edges are inserted to or deleted from the graph in a way to minimize the overall costs.

#### EM clustering (EM)

EM Clustering [14] is an EM-type algorithm [19] that estimates consensus peaks by clustering the peaks detected by peak picking. It is assumed that the peaks can be interpreted as observations of an underlying Gaussian model of the consensus peaks, with known standard deviation in both dimensions. Alternatingly, weights and parameters are optimized. In contrast to the original EM algorithm with a fixed number of clusters, a merging step is included within each EM-iteration, allowing to merge consensus peaks.

### Classification methods

We use a wide range of well-established classification algorithms, based on the estimation of decision boundaries (*Support Vector Machine*), based on similarities of observations (*K-Nearest-Neighbor*) and tree-based algorithms (*Classification Tree*, *Generalized Boosted Models*, *Random Forest*). For information about the particular algorithms see [20]. Classification was performed using R version 3.2.2 and the packages e1071 (SVM), kknn (kNN), rpart (CT), gbm (GBM), and randomForest (RF).

A 10-fold cross-validation (CV) is performed. The initial dataset is divided into 10 equally sized groups, each balanced such that the percentage of patients and healthy controls is nearly the same as in the entire dataset. The classification rule is then based on nine of the ten groups (training set) and the performance of the classification is evaluated on the left out tenth group (test set). This is repeated ten times such that each group and thus each observation is evaluated exactly once during one CV run. Since the results depend on the initial random split into ten groups, the CV is repeated 50 times, increasing the stability of the results and assessing the variability of the algorithms.

For algorithms with parameter tuning a nested CV (consisting of outer and inner CV) is performed, see supplemental material (S1 Inf) for details on parameter settings and tuning. The (outer) training set is split into ten groups as described above. The inner training set is used to optimize the algorithm parameters, and classification accuracy is tested on the inner test set. Parameters are selected that perform best on the ten inner test sets. These parameters are used to create the classifier based on the outer training set. In the following, we briefly describe all utilized classification methods.

#### Support vector machine (SVM)

The Support Vector Machine searches for decision boundaries such that the observations of each class are on one side of the boundary and have as much distance to it as possible. A simple classification boundary is a hyperplane. For a *Linear Support Vector Machine* the hyperplane is fitted such that the target function, i.e. the sum of the distances of the observations to the boundary, is maximized. Observations on the wrong side of the boundary lead to a penalization in the target function. Nonlinear boundaries can be achieved by transforming the observations to a higher dimension and fitting there a linear hyperplane. Using a kernel function (here a radial basis kernel) allows solving the optimization problem directly, based only on distances between the transformed observations.

#### K-Nearest-Neighbor (kNN)

A simple and intuitive approach for classification is k-Nearest-Neighbor classification. An observation from the test set is assigned to the class to which most of its *k* closest neighbors belong (majority vote, ties are randomly assigned to one of the classes). Distance is measured by Euclidean distance.

#### Classification tree (CT)

The Classification Tree iteratively splits the observations into two classes using one variable at a time. In the beginning, all observations are in one so-called “node”. The best variable and its appropriate split point are chosen according to an impurity measure (here the Gini Index) that evaluates the success of a certain split. Resulting nodes with observations of just one class are desirable. Each node can be further split into two groups until perfect separation or a stopping criterion (tree depth, minimum number of observations in a node for splitting or a terminal node, no sufficient decrease of the Gini Index, etc.) is reached.

#### Generalized boosted models (GBM)

GBM is an extension of the single Classification Tree. It is a boosting approach using a differentiable loss function (here binomial deviance) to measure the difference between the predicted class probabilities and the true labels. The expected negative gradient of the loss function is modeled by a Regression Tree, based on a subsample of 50% of the observations. The current decision function is updated by adding the predictions in the terminal nodes of the regression tree. This is repeated until a fixed number of iterations (trees) is reached.

#### Random forest (RF)

The Random Forest is also an extension of the single Classification Tree. Its classification rule is a majority vote of many Classification Trees (here 500). Since low correlation between trees is desired, each tree is based on a bootstrap sample of the original sample size and for each split only $\sqrt{p}$ randomly chosen out of all *p* variables are considered. Trees are grown to the maximum size (without restrictions on the terminal node size).

### Study design

In an exhaustive study design, we apply all feasible combinations of peak picking, peak clustering, and classification algorithms to the three datasets introduced in Section Data. Most algorithms can be combined, in total 156 combinations (26 methods for peak definition including VN^*m*^ and 6 classification algorithms) were applied.

Due to technical limitations, VN^*a*^ peak clustering cannot be combined with other peak picking methods, but the peaks found by VN^*a*^ peak picking can be used as input for other peak clustering methods. Also the manual approach VN^*m*^ does not separate peak picking and peak clustering, thus no automated algorithm can be combined with this manual technique.

A 10-fold cross-validation (CV) is applied in order to achieve unbiased AUC (area under the ROC curve) values. In comparison to accuracy as a performance measure, the proportion of all correct classifications, the AUC is a trade-off measure between true positive rate (the proportion of *true positives* among all observations assigned to the positive class) and the false positive rate (the proportion of *false positives* among all observations assigned to the positive class). The disadvantage of using accuracy is that it is dependent on the probability cutpoint for assigning observations to the two classes and that in case of unbalanced classes the larger group is favored by the model. Each CV is performed 50 times and medians across these 50 replications are reported unless stated differently.

## Results

We use AUC values obtained in two-class classification tasks as quality measures for our pipelines. We first analyze which combinations of peak finding, peak clustering, and classification algorithm yield competitive results on all three datasets. To address a potential overfitting due to the large number of algorithms tested, we then evaluate the three steps separately with respect to their overall performance and the stability of the resulting performance independent of the other steps.

Table 1 shows for each dataset the first quantile, the median and the third quartile of AUC values over all pipelines and the 50 replicates. We see that the three classification tasks have different levels of difficulty. The median values are 0.933, 0.827, and 0.721, and the interquartile ranges are varying between 0.09 and 0.15, which together implies relevant differences between the tasks.

**Table 1:** Quartiles of performance for all datasets over all combinations of peak picking, clustering, statistical classification, and all replications of the cross-validation.

	1st Qu.	Median	3rd Qu.
*D*_1_	0.878	0.933	0.965
*D*_2_	0.780	0.827	0.874
*D*_3_	0.643	0.721	0.792

### Analysis of overall pipelines

The main interest is to find the best combination of peak picking, peak clustering, and classification algorithm that can be used as fully automated processing procedure.

For each dataset and the 156 pipelines, the median AUC values of the 50 CV replications were ordered. For each pipeline, its corresponding three ranks were summed up. Table 2 shows the top 20 pipelines ordered by their rank sum (RS). In addition to the rank sum, the arithmetic mean of the three median AUCs obtained on the different datasets are listed.

**Table 2:** Ranks of median AUCs for each combination of peak picking, peak clustering and classification algorithms and ranksum over the three datasets and corresponding mean AUC.

				Rank			
Peak	Cluster	Classif	*AUC*	*D*_1_	*D*_2_	*D*_3_	RS
SGLTR	DBSCAN	RF	0.957	12	3	1	16
SGLTR	EM	RF	0.950	12	2	5	19
SGLTR	CE	RF	0.947	14	7	3	24
VN^*m*^	VN^*m*^	RF	0.936	6	19	2	27
LM	DBSCAN	RF	0.916	2	15	18	35
VN^*a*^	VN^*a*^	RF	0.925	3	9	23	35
LM	EM	RF	0.913	5	11	26	42
VN^*a*^	DBSCAN	RF	0.919	8	31	4	43
SGLTR	EM	GBM	0.927	26	5	15	46
LM	GS	RF	0.914	4	4	44	52
SGLTR	GS	RF	0.907	17	12	32	61
SGLTR	CE	GBM	0.919	48	7	16	71
VN^*a*^	VN^*a*^	SVM^rbf^	0.897	27	27	20	74
VN^*a*^	EM	RF	0.889	1	60	17	78
VN^*a*^	DBSCAN	GBM	0.914	50	20	10	80
OPME	DBSCAN	RF	0.885	15	46	29	90
VN^*m*^	VN^*m*^	GBM	0.901	31	51	8	90
LM	DBSCAN	GBM	0.884	18	13	62	93
SGLTR	DBSCAN	GBM	0.921	82	6	11	99
VN^*a*^	CE	RF	0.894	33	54	12	99

The best combination of algorithmic steps is SGLTR peak picking with DBSCAN peak clustering and RF classification, with ranks 12, 3, and 1 on the three datasets. The second and third best combination differ only in peak clustering (EM and CE), but also contain SGLTR and RF. Rank 4 is occupied by the current gold standard VN^*m*^ that performs better than the best combination on the first dataset (rank 6) but worse on the others (rank 19 and 2, respectively).

**Fig 2:**
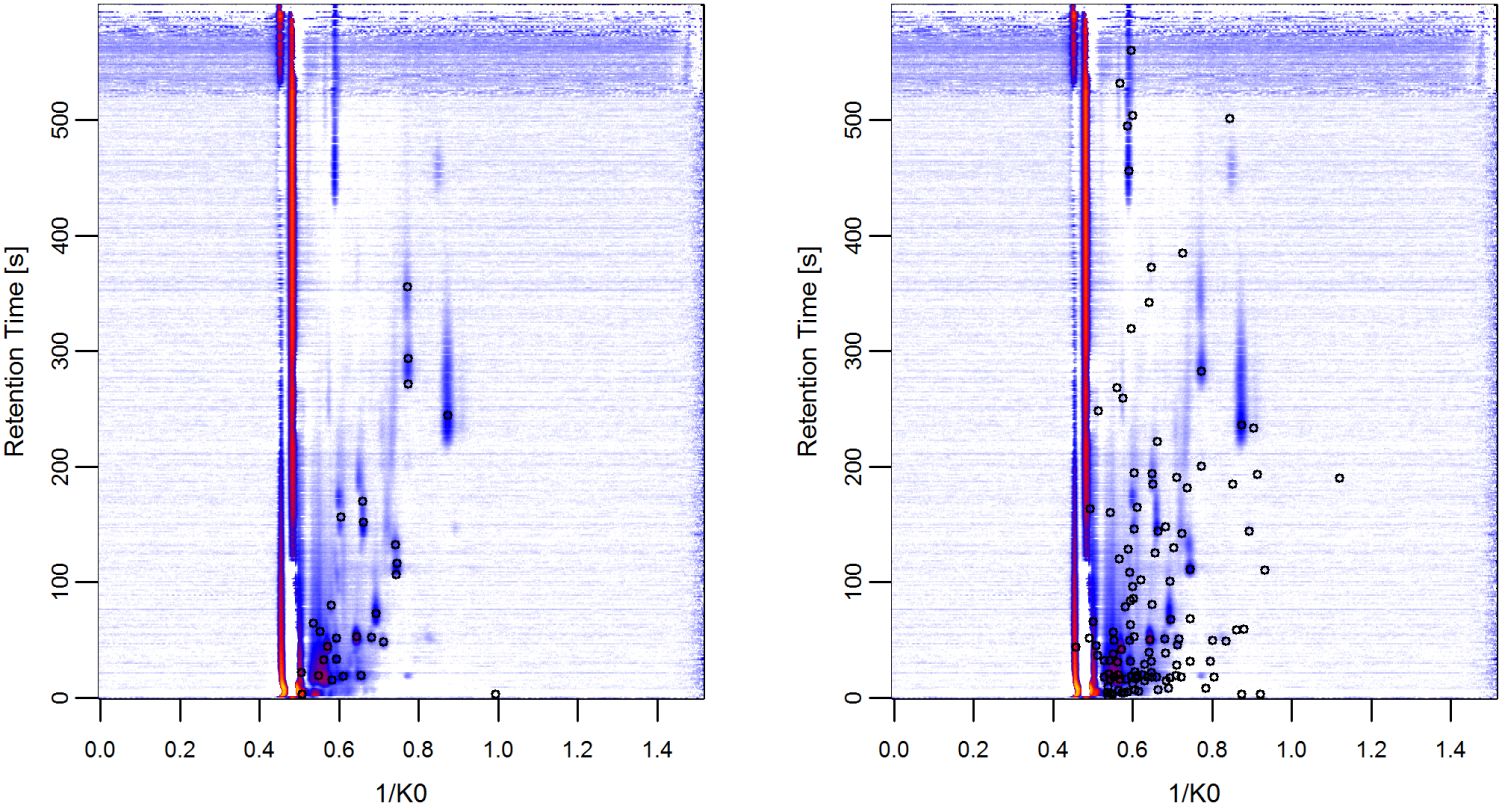
Averaged raw measurements of the first dataset and the consensus peaks identified by the combination of SGLTR/DBSCAN (left) and the manual gold standard VN^*m*^ (right).

In Fig 2 we display the comparison of the peaks identified by the combination of SGLTR/DBSCAN and VN^*m*^ for the first dataset. The underlying image is the average of all raw measurements of this dataset, so the consensus peaks should ideally lie above the visually spotted peaks. As we can see (on the left), the peaks identified by the automated algorithm cover most but not all peaks that can be spotted visually. Instead, VN^*m*^ (on the right) also detects peaks that can not be seen in this data representation (based on average measurements).

In the classification step RF clearly achieves the best results. Combinations in the top 20 table using other classification algorithms (six times GBM and once SVM^rbf^) always perform better when the classification step is replaced with RF.

SGLTR peak picking was combined twice with DBSCAN, EM, and CE, and once with GS. Besides SGLTR and VN^*m*^ also LM and VN^*a*^ occur in the table, mostly with EM or DBSCAN clustering (VN^*a*^ also with its own peak clustering). The peak clustering method seems to be of lower importance since it varies a lot with similar performance, whereas the choices of classification and peak picking methods have a clear impact.

More detailed information can be obtained from S1 Fig to S3 Fig in the supplemental material. These visualize all AUCs of all pipelines on the three datasets. For each pipeline, boxplots of the 50 AUC values across replications are shown. Each graphic contains six panels corresponding to the six classification algorithms, whereas peak picking methods are indicated on the x-axis, and peak clustering algorithms by color.

We observe that RF mostly achieves higher median AUCs than the other classification algorithms with less variation, the latter probably because no parameter tuning is required for RF. We also observe differences in peak picking and peak clustering methods but it is hard to find a pattern visually. In the following subsections we analyze the impact of all single steps in detail.

### Evaluation of single steps in the pipelines

When evaluating the three analysis steps separately, one has to consider the potentially strong dependence between peak picking and peak clustering. A sensitive peak picking method that finds many peaks might require a rather tight clustering. In contrast, the influence of the classification step is assumed to be rather independent of the first two steps.

In the following, we average AUC values for a fixed algorithm in one step over all possible choices in the other two steps. We are aware that the best combinations might be in disagreement with the results, but our goal is to quantify the general impact of single algorithmic steps and to avoid over-interpretation. Looking only at the best combination might lead to overoptimistic evaluations due to the large number of evaluated pipelines.

#### Classification

Table 2 with the 20 best performing pipelines indicates a greater impact of the choice of the classification algorithm, compared to the other two steps. Table 3 shows the median AUCs for all classification algorithms across all peak picking and clustering methods, separately for each dataset. Indeed, RF classification outperforms the alternatives on all datasets. GBM always ranks second and kNN always last. The difference of medians between RF and kNN ranges from 0.088 for the first and 0.144 for the third dataset which are meaningful differences in terms of AUC.

**Table 3:** Performance for all classification algorithms over all peak picking and peak clustering algorithms and all replications for each dataset.

	SVM^lin^	SVM^rbf^	kNN	CT	GBM	RF
*D*_1_	0.937	0.935	0.889	0.863	0.939	0.977
*D*_2_	0.808	0.806	0.789	0.819	0.874	0.890
*D*_3_	0.727	0.735	0.664	0.685	0.742	0.808

#### Peak picking

For peak picking, we first analyze and compare the numbers of detected peaks. In Table 4 we report the median, minimum, and maximum of identified peaks in individual measurements for every dataset separately. For VN^*m*^ with combined peak picking and clustering, no numbers can be given. The other numbers vary from zero to 143, with largest medians 42 (SGLTR, first dataset *D*_1_), 49 (SGLTR, *D*_2_), and 56 (VN^*a*^, *D*_3_). Variation across datasets is high, with largest numbers for *D*_2_. SGLTR is the only algorithm detecting at least 15 peaks in every measurement. Some algorithms lead to implausible results, for example PDSA that finds for *D*_2_ as median only seven peaks per measurement, with minimum zero and maximum 90. The third dataset leads to the most stable results with lowest variability and with at least 19 detected peaks for all measurements and all peak picking methods.

**Table 4:** Number of peaks detected by each peak picking method in the single measurements.

	LM	PME	PDSA	SGLTR	OPME	VN^*a*^	VN^*m*^
*D*_1_							
Median	13	14	15	42	31	30	–
Min	5	5	3	15	5	0	–
Max	34	54	47	115	69	62	–
*D*_2_							
Median	12	17	7	49	14	10	–
Min	5	6	0	15	3	0	–
Max	69	69	90	137	61	143	–
*D*_3_							
Median	29	32	33	45	29	56	–
Min	20	23	22	34	19	25	–
Max	38	53	46	84	41	93	–

In Table 5 we summarize classification performance of peak picking methods, calculating median values across all other steps (peak clustering, classification and replications). VN^*m*^ achieves the best results with ranks (2, 1, 1) for the three datasets. The best automated peak picking methods are LM with ranks (1, 2, 5) and SGLTR with ranks (3, 4, 2). Restricting the classification step to the best performing RF (see Table S1 Table in the supplemental material), the rankings do not change much (VN^*m*^ has ranks (2, 3, 1), LM (1, 2, 5), and SGLTR (4, 1, 2)), but almost all median AUC values are clearly higher than before.

**Table 5:** Performance for all peak picking algorithms over all peak clustering and classification algorithms and all replications for each dataset.

	LM	PME	PDSA	SGLTR	OPME	VN^*a*^	VN^*m*^
*D*_1_	0.959	0.880	0.940	0.948	0.891	0.925	0.955
*D*_2_	0.851	0.812	0.811	0.839	0.798	0.843	0.874
*D*_3_	0.731	0.587	0.664	0.777	0.741	0.764	0.828

#### Peak clustering

After identifying peaks in all individual measurements, peak clustering determines which peaks are likely to be caused by the same metabolite. We call these final (clustered) peaks “features”. Table 6 contains the numbers of features for all combinations and for each dataset. Note that VN^*m*^ combines both steps and cannot be mixed with other algorithms and that peak clustering of VN^*a*^ cannot be applied after other peak finding methods.

**Table 6:** Numbers of consensus peaks detected by each combination of peak picking and peak clustering, summarized over all measurements in a dataset.

		LM	PME	PDSA	SGLTR	OPME	VN^*a*^	VN^*m*^
*D*_1_	GS	42	51	43	138	124	112	–
	DBSCAN	25	26	26	28	66	29	–
	CE	26	23	22	52	35	25	–
	EM	42	56	44	142	116	98	–
	VN^*a*^	–	–	–	–	–	239	–
	VN^*m*^	–	–	–	–	–	–	120
*D*_2_	GS	23	25	17	46	11	20	–
	DBSCAN	16	14	23	43	16	30	–
	CE	18	23	26	66	35	37	–
	EM	19	26	27	67	19	51	–
	VN^*a*^	–	–	–	–	–	239	–
	VN^*m*^	–	–	–	–	–	–	224
*D*_3_	GS	62	53	62	75	54	132	–
	DBSCAN	40	23	43	52	42	56	–
	CE	34	25	35	54	38	34	–
	EM	47	57	56	59	56	105	–
	VN^*a*^	–	–	–	–	–	265	–
	VN^*m*^	–	–	–	–	–	–	60

In general, VN^*a*^ peak picking generates most features, especially when combined with its corresponding peak clustering. The current gold standard VN^*m*^ creates 120, 60, and 224 features on the three datasets, respectively. These strongly differing numbers are partly due to the manual peak labeling which was done at the same time for all measurements of a data source (e.g. a hospital), not only for the specific datasets analyzed in this study. Thus, additional peaks that are not observed in the analyzed datasets can be included.

Since peak picking and clustering are inherently connected we do not analyze the impact of peak clustering on its own, but only in combination with peak picking. In Table 7, all 26 combinations of peak picking and clustering are listed. To find the best clustering method for each peak picking algorithm we calculate two measures. The first one is the median AUC over all classification methods and its replications. For the second one, median AUC values were calculated separately for each combination of classification algorithm, each peak picking and peak clustering method. Then ranks were calculated among the clustering methods to find the best one for each setting of classification and peak picking. For VN^*a*^ five peak cluster algorithms are available, for all others four which makes those numbers the maximum ranks. VN^*m*^ does not have clustering methods and thus is neglected in this comparison. Afterwards, all ranks for a certain combination of peak picking and clustering were summed up over the six classification algorithms and divided by the minimum rank sum (which is six when one combination was the best in all classification algorithms). The result is the mean rank sum. It has values between one, when a peak clustering method outperforms all others independent of the classification method, and four (or five for VN^*a*^). This way we can assess how often a combination outperforms the others.

**Table 7:** Median AUCs over all replications of each classification algorithm and rank sum of median AUCs for each classification algorithm.

		*D*_1_		*D*_2_		*D*_3_	
Picking	Clustering	AUC	RS	AUC	RS	AUC	RS
LM	GS	0.957	2.67	0.868	2.50	0.662	3.67
LM	DBSCAN	0.970	1.50	0.825	2.50	0.736	2.50
LM	CE	0.926	4.00	0.808	3.33	0.742	2.17
LM	EM	0.970	1.83	0.873	1.67	0.749	1.67
PME	GS	0.868	2.67	0.836	2.00	0.637	1.50
PME	DBSCAN	0.945	1.00	0.825	2.83	0.568	3.00
PME	CE	0.802	3.50	0.781	3.33	0.561	2.83
PME	EM	0.875	2.83	0.833	1.83	0.568	2.67
PDSA	GS	0.928	2.83	0.802	3.17	0.586	3.33
PDSA	DBSCAN	0.965	1.83	0.803	3.00	0.729	2.17
PDSA	CE	0.879	4.00	0.817	2.00	0.644	2.83
PDSA	EM	0.977	1.33	0.817	1.83	0.720	1.67
SGLTR	GS	0.936	3.50	0.822	3.50	0.724	3.50
SGLTR	DBSCAN	0.963	2.25	0.827	2.83	0.857	1.17
SGLTR	CE	0.932	3.17	0.859	1.83	0.800	2.33
SGLTR	EM	0.971	1.08	0.840	1.83	0.777	3.00
OPME	GS	0.897	2.83	0.699	4.00	0.762	1.83
OPME	DBSCAN	0.943	1.17	0.812	2.33	0.716	3.00
OPME	CE	0.818	3.83	0.817	1.67	0.752	1.83
OPME	EM	0.904	2.17	0.829	2.00	0.713	3.33
VN^*a*^	GS	0.904	4.50	0.793	4.50	0.697	4.33
VN^*a*^	DBSCAN	0.924	2.83	0.876	1.83	0.829	2.00
VN^*a*^	CE	0.897	3.67	0.852	3.00	0.786	3.17
VN^*a*^	EM	0.947	2.67	0.815	3.83	0.753	2.83
VN^*a*^	VN^*a*^	0.962	1.33	0.892	1.83	0.774	2.67
VN^*m*^	VN^*m*^	0.955	1.00	0.874	1.00	0.828	1.00

For some peak picking algorithms there is a clearly preferable peak clustering method. LM and PDSA work best when combined with EM, and VN^*a*^ with its own clustering method or with DBSCAN. For PME, GS or DBSCAN achieve best results. For SGLTR and OPME the best combination depends on the dataset. For SGLTR all cluster methods except GS dominate the others on one dataset, for OPME it is even more divers.

## Summary and conclusion

Broad applicability of MCC-IMS is still hampered by the current need for manual intervention during peak assignment on the raw measurements. We analyzed the entire process from raw MCC-IMS measurements towards disease classification and compared several up-to-date automatic algorithms. In total, 25 automated peak finding methods (all feasible combinations of 6 peak picking and 5 peak clustering algorithms) are compared to the current gold standard that requires manual supervision. After the feature definition, statistical classification was performed by six different classification algorithms. In total, 156 comparisons were made, thereof 150 fully automated.

We aimed at finding the best pipeline, i.e. the best triple of peak picking, peak clustering and classification algorithm, and at gaining further insight into the stability of this choice. For this task we used AUC values on three different real datasets to assess the performance of the three steps. Accordingly, peak picking and clustering were considered successful when the resulting AUC values of the final classification was high.

Specifically we showed that the classification algorithm has the greatest impact on the performance, with RF (Random Forest) clearly outperforming the other methods. The best peak picking methods in our study were LM (Local Maxima) and SGLTR (Savitzky-Golay Laplace-operator filtering thresholding Regions). For peak clustering it was not always clear which method fits best to the peak picking algorithms, but mostly DBSCAN (Density-Based Spatial Clustering of Applications with Noise) and EM achieved the best results. Looking at all possible combinations, the combination of SGLTR peak picking, DBSCAN peak clustering and RF classification performed best, in agreement with the individual results. We recommend to use this combination for future automated peak detection and classification analysis.

However, we cannot conclude that SGLTR and DBSCAN are the best algorithms for the peak definition task itself, since we only judge them by the ability to classify based on the detected peaks. Thus, it is possible that these algorithms do not find all relevant peaks in each measurement but that the multivariate classification approach compensates for possible weaknesses. This is no serious issue in a new application where it is unknown which compounds in the air show differences in the groups. Once these analytes are identified based on the classification results, further research should be carried out in order to develop an algorithm tailored to a specific application ensuring that all relevant peaks are detected in each measurement.

To conclude, several combinations of automated algorithms keep up with the current manual gold standard or even yield better results. This shows that the tedious, error-prone and subjective manual preprocessing can be fully replaced by automated algorithms, an important step to make the whole technological approach applicable also in large studies or for various applications.

## Supporting information

S1 Inf Information about tuning of classification algorithms. Information about the R packages used for statistical classification and the parameters optimized in nested cross-validation.
(PDF)

S1 Fig Detailed AUCs for the first dataset. AUC values for all combinations of peak picking, peak clustering, and classification methods for the first dataset. Each box represents the cross-validated AUCs for the 50 replications. The horizontal line at AUC = 0.9 simplifies comparisons between the panels.
(EPS)

S2 Fig Detailed AUCs for the second dataset. AUC values for all combinations of peak picking, peak clustering, and classification methods for the second dataset. Each box represents the cross-validated AUCs for the 50 replications. The horizontal line at AUC = 0.9 simplifies comparisons between the panels.
(EPS)

S3 Fig Detailed AUCs for the third dataset. AUC values for all combinations of peak picking, peak clustering, and classification methods for the third dataset. Each box represents the cross-validated AUCs for the 50 replications. The horizontal line at AUC = 0.9 simplifies comparisons between the panels.
(EPS)

S1 Table Performance for all peak picking algorithms over all peak clustering and all replications for each dataset, using RF classification. (PDF)

S2 Table Median AUC values for combinations of peak picking and peak clustering for RF classification. (PDF)

## References

[1] Cao W, Duan Y. Breath Analysis: Potential for Clinical Diagnosis and Exposure Assessment. Clinical Chemistry. 2006;52(5):800–811. 10.1373/clinchem.2005.063545 16513771

[2] Fink T, Baumbach JI, Kreuer S. Ion mobility spectrometry in breath research. Journal of Breath Research. 2014;8(2):027104 10.1088/1752-7155/8/2/027104 24682214

[3] Horsch S, Kopczynski D, Baumbach JI, Rahnenführer J, Rahmann S. From raw ion mobility measurements to disease classification: a comparison of analysis processes. PeerJ PrePrints. 2015;3:e1591.

[4] Westhoff M, Litterst P, Maddula S, Bödeker B, Rahmann S, Davies AN, et al Differentiation of chronic obstructive pulmonary disease (COPD) including lung cancer from healthy control group by breath analysis using ion mobility spectrometry. International Journal for Ion Mobility Spectrometry. 2010;13(3-4):131–139. 10.1007/s12127-010-0049-2

[5] Rabis T, Sommerwerck U, Anhenn O, Darwiche K, Freitag L, Teschler H, et al Detection of infectious agents in the airways by ion mobility spectrometry of exhaled breath. International Journal for Ion Mobility Spectrometry. 2011;14(4):187–195. 10.1007/s12127-011-0077-6

[6] Bader S, Urfer W, Baumbach JI. Preprocessing of ion mobility spectra by lognormal detailing and wavelet transform. International Journal for Ion Mobility Spectrometry. 2008;11(1-4):43–49. 10.1007/s12127-008-0005-6

[7] Savitzky A, Golay MJE. Smoothing and differentiation of data by simplified least squares procedures. Analytical chemistry. 1964;36(8):1627–1639. 10.1021/ac60214a047

[8] Bödeker B, Vautz W, Baumbach JI. Visualisation of MCC/IMS-data. International Journal for Ion Mobility Spectrometry. 2008;11(1-4):77–81. 10.1007/s12127-008-0011-8

[9] Bader S, Urfer W, Baumbach JI. Processing ion mobility spectrometry data to characterize group differences in a multiple class comparison. International Journal for Ion Mobility Spectrometry. 2005;8:1–4.

[10] D’Addario M, Kopczynski D, Baumbach JI, Rahmann S. A modular computational framework for automated peak extraction from ion mobility spectra. BMC Bioinformatics. 2014;15(1):25 10.1186/1471-2105-15-25 24450533 PMC3930762

[11] Kopczynski D, Baumbach JI, Rahmann S. Peak modeling for Ion mobility spectrometry measurements. In: Signal Processing Conference (EUSIPCO), 2012 Proceedings of the 20th European. New York, NY, USA: IEEE; 2012. p. 1801–1805.

[12] Egorov A, König A, Köppen M, Kühn H, Kullack I, Kuthe E, et al. Ressourcenbeschränkte Analyse von Ionenmobilitätsspektren mit dem Raspberry Pi. Faculty of computer science, TU Dortmund; 2014.

[13] Hauschild AC, Kopczynski D, D’Addario M, Baumbach JI, Rahmann S, Baumbach J. Peak Detection Method Evaluation for Ion Mobility Spectrometry by Using Machine Learning Approaches. Metabolites. 2013;3(2):277–293. 10.3390/metabo3020277 24957992 PMC3901270

[14] Kopczynski D, Rahmann S. An online peak extraction algorithm for ion mobility spectrometry data. Algorithms for Molecular Biology. 2015;10(1):17 10.1186/s13015-015-0045-5 26157473 PMC4495807

[15] Bödeker B, Vautz W, Baumbach JI. Peak finding and referencing in MCC/IMS-data. International Journal for Ion Mobility Spectrometry. 2008;11(1):83–87.

[16] Ester M, Kriegel HP, Sander J, Xu X. A density-based algorithm for discovering clusters in large spatial databases with noise. In: Knowledge Discovery and Data Mining (KDD), Proceedings of first international conference. vol. 96; 1996. p. 226–231.

[17] Rahmann S, Wittkop T, Baumbach J, Martin M, Truss A, Böcker S. Exact and heuristic algorithms for weighted cluster editing. In: Computational Systems Bioinformatics Conference. vol. 6; 2007. p. 391–401. 17951842

[18] Böcker S, Briesemeister S, Klau GW. Exact algorithms for cluster editing: Evaluation and experiments. Algorithmica. 2011;60(2):316–334. 10.1007/s00453-009-9339-7

[19] Dempster AP, Laird NM, Rubin DB. Maximum likelihood from incomplete data via the EM algorithm. Journal of the Royal Statistical Society Series B (Methodological). 1977;39(1):1–38.

[20] Hastie T, Tibshirani R, Friedman J. The Elements of Statistical Learning, Second Edition New York, USA: Springer Series in Statistics; 2009.

