# Supplementary material for: A detailed comparison of analysis processes for MCC-IMS data in disease classification—Automated methods can replace manual peak annotations
Source: PLoS One. 2017 Sep 14;12(9):e0184321. doi: 10.1371/journal.pone.0184321 (PMC5598980; doi:10.1371/journal.pone.0184321)
Supplement: S1 Inf — Information about the R packages used for statistical classification and the parameters optimized in nested cross-validation. (PDF) [file pone.0184321.s001.pdf]

Information on parameters used by the classification algorithms

Classification was performed using R [1] version 3.2.2. The used parameters (where different from the default settings) are listed below. When parameter optimization (grid search) was performed, the considered values are given. Often a wide range of possible values is considered on an exponential grid 2^x where x takes the 10 equidistant values between -15 and 15. We refer to this set as "exponential grid".

Support Vector Machine (SVM)

- **R-package:** e1071 [2]
- **Parameters:**
  - `cost`: optimization on the exponential grid
  - `kernel`: "linear" (for linear SVM) and "radial" for rbf SVM
  - `gamma`: optimization on the exponential grid (just for rbf SVM)

K-Nearest-Neighbor (kNN)

- **R-package:** kknn [3]
- **Parameters:**
  - `k`: optimization on 1, 2, ..., 10

Classification Tree (CT)

- **R-package:** rpart [4]
- **Parameters:**
  - `minbucket`: optimization on 1, 2, ..., 5

Generalized Boosted Models (GBM)

- **R-package:** gbm [5]
- **Parameters:**
  - `shrinkage`: optimization on the exponential grid
  - `n.minobsinnode`: optimization on 1, 2, ...5
  - `interaction.depth`: optimization on 1, 2, 3

Random Forest (RF)

- **R-package:** randomForest [6]
- no parameter optimization

References

1. R Core Team. R: A Language and Environment for Statistical Computing; 2016. Available from: https://www.R-project.org/.

2. Meyer D, Dimitriadou E, Hornik K, Weingessel A, Leisch F. e1071: Misc Functions of the Department of Statistics (e1071), TU Wien; 2014. Available from: http://CRAN.R-project.org/package=e1071.

3. Schliep K, Hechenbichler K. kknn: Weighted k-Nearest Neighbors; 2014. Available from: http://CRAN.R-project.org/package=kknn.

4. Therneau T, Atkinson B, Ripley B. rpart: Recursive Partitioning and Regression Trees; 2015. Available from: http://CRAN.R-project.org/package=rpart.

5. Ridgeway G, et al.. gbm: Generalized Boosted Regression Models; 2013. Available from: http://CRAN.R-project.org/package=gbm.

6. Liaw A, Wiener M. Classification and Regression by randomForest. R News. 2002;2(3):18–22.
