# Supplementary material for: A detailed comparison of analysis processes for MCC-IMS data in disease classification—Automated methods can replace manual peak annotations
Source: PLoS One. 2017 Sep 14;12(9):e0184321. doi: 10.1371/journal.pone.0184321 (PMC5598980; doi:10.1371/journal.pone.0184321)
Supplement: S1 Table — (PDF) [file pone.0184321.s005.pdf]

	LM	PME	PDSA	SGLTR	OPME	VN^a	VN^m
D_1	0.988	0.956	0.967	0.980	0.962	0.983	0.985
D_2	0.934	0.904	0.809	0.973	0.856	0.856	0.922
D_3	0.811	0.685	0.738	0.889	0.815	0.838	0.899
