# Supplementary material for: A detailed comparison of analysis processes for MCC-IMS data in disease classification—Automated methods can replace manual peak annotations
Source: PLoS One. 2017 Sep 14;12(9):e0184321. doi: 10.1371/journal.pone.0184321 (PMC5598980; doi:10.1371/journal.pone.0184321)
Supplement: S2 Table — (PDF) [file pone.0184321.s006.pdf]

	GS	DBSCAN	CE	EM	VN^a	VN^m
D_1						
LM	0.989	0.991	0.951	0.986	–	–
PME	0.953	0.974	0.941	0.958	–	–
PDSA	0.952	0.978	0.926	0.985	–	–
SGLTR	0.977	0.981	0.979	0.981	–	–
OPME	0.949	0.978	0.925	0.972	–	–
VN^a	0.949	0.983	0.969	0.994	0.991	–
VN^m	–	–	–	–	–	0.985
D_2						
LM	0.975	0.929	0.926	0.939	–	–
PME	0.985	0.890	0.887	0.915	–	–
PDSA	0.778	0.819	0.821	0.810	–	–
SGLTR	0.935	0.979	0.968	0.981	–	–
OPME	0.776	0.864	0.866	0.853	–	–
VN^a	0.832	0.883	0.856	0.841	0.963	–
VN^m	–	–	–	–	–	0.922
D_3						
LM	0.776	0.829	0.821	0.815	–	–
PME	0.722	0.668	0.672	0.689	–	–
PDSA	0.707	0.739	0.737	0.788	–	–
SGLTR	0.809	0.910	0.894	0.887	–	–
OPME	0.810	0.813	0.846	0.800	–	–
VN^a	0.769	0.890	0.858	0.833	0.822	–
VN^m	–	–	–	–	–	0.899
